# Reverse genetics in the *Arabidopsis* chloroplast genome identifies *rps16* as a transcribed pseudogene

**DOI:** 10.1111/tpj.70198

**Published:** 2025-05-07

**Authors:** Stephanie Ruf, Raphael Trösch, Laura Schollbach, Xenia Kroop, Joachim Forner, Shany Gefen‐Treves, Anita Henze, Wolfram Thiele, Mark A. Schöttler, Reimo Zoschke, Ralph Bock

**Affiliations:** ^1^ Max‐Planck‐Institut für Molekulare Pflanzenphysiologie Am Mühlenberg 1 D‐14476 Potsdam‐Golm Germany; ^2^ Present address: Faculty of Biology Rheinland‐Pfälzische Technische Universität Kaiserslautern‐Landau 67663 Kaiserslautern Germany

**Keywords:** plastid, plastid transformation, ribosomal protein, translation, evolution, pseudogene, gene transfer

## Abstract

The plastid (chloroplast) genomes of seed plants contain a conserved set of ribosomal protein genes. The *rps16* gene represents an exception: It has been lost from the plastid genomes of gymnosperms and several lineages of angiosperms, and may have undergone pseudogenization in a few other lineages, including members of the Brassicaceae family. Here we report a reverse genetic approach to test the annotated *rps16* gene in the *Arabidopsis* plastid genome for functionality. Employing the recently developed plastid transformation technology for the model plant *Arabidopsis*, we have deleted the putative *rps16* gene from the *Arabidopsis* plastid genome. We report that the resulting transplastomic plants display wild‐type‐like growth and photosynthetic performance under a wide range of conditions. Moreover, genome‐wide analyses of chloroplast transcript levels and ribosome footprints revealed unaltered plastid translational activity in Δrps16 mutants compared with wild‐type plants. We conclude that the annotated *rps16* gene in the plastid genome of *Arabidopsis* is a transcribed pseudogene that has been replaced in evolution by a nuclear gene copy that supplies functional S16 protein to chloroplasts.

## Significance Statement

This study applied the recently developed plastid transformation technology for the model plant *Arabidopsis* to investigate the function of *rps16*, a non‐conserved ribosomal protein gene in seed plant chloroplast genomes. The results reveal that *rps16* is a plastid pseudogene, and functional Rps16 protein is supplied by a nuclear gene copy.

## INTRODUCTION

Translation in plastids (chloroplasts) occurs on bacterial‐type 70S ribosomes that are similar in structure, composition, and function to bacterial ribosomes (Bieri et al., 2017; Perez Boerema et al., 2018; Tiller & Bock, 2014; Zoschke & Bock, 2018). All four RNA components (the ribosomal RNAs, rRNAs) of the plastid ribosome are encoded in the chloroplast genome: the 23S, 5S, and 4.5S rRNAs in the large (50S) ribosomal subunit, and the 16S rRNA in the small (30S) ribosomal subunit. By contrast, the protein constituents of the chloroplast ribosome, the plastid ribosomal proteins, are only partly encoded in the chloroplast genome, and a large fraction of them is encoded by nuclear genes, translated on cytosolic (80S) ribosomes, and post‐translationally imported into plastids. Of the core set of ribosomal proteins (identified by their homology to ribosomal proteins from *Escherichia coli*), 21 proteins are encoded in the plastid genome of the model plant tobacco (*Nicotiana tabacum*), whereas the remaining 31 are encoded in the nucleus. In addition to these conserved prokaryotic ribosomal proteins, plastid ribosomes also contain a small number of proteins that are not present in the *E. coli* ribosome. These so‐called plastid‐specific ribosomal proteins (PSRPs) all are encoded in the nucleus. Their functions in the translation process, in ribosome assembly, and/or in translational regulation are currently not well understood (Tiller et al., 2012; Tiller & Bock, 2014).

In dicots, plastid translational activity is essential for cell survival (Ahlert et al., 2003), whereas in a few monocots (grasses), mutants devoid of plastid ribosomes have been described. They have severe phenotypes and do not survive beyond the seedling stage (Han et al., 1992; Hess et al., 1993). Reverse genetic studies in tobacco chloroplasts have revealed that the plastid translational apparatus contains a few nonessential components, including a small number of ribosomal proteins and tRNAs (Alkatib et al., 2012b; Rogalski, Karcher, & Bock, 2008; Fleischmann et al., 2011), whereas all rRNAs and the vast majority of ribosomal proteins and tRNAs are indispensable for plant viability (Alkatib et al., 2012a; Rogalski et al., 2006; Rogalski, Karcher, & Bock, 2008; Rogalski, Schöttler, et al., 2008). Similarly, disruption of essential nucleus‐encoded plastid ribosomal proteins results in embryo lethality in dicotyledonous plants (Romani et al., 2012; Tiller & Bock, 2014; Yin et al., 2012).

The set of ribosomal protein genes present in the chloroplast genome is generally well conserved in vascular plants, indicating that functional gene transfer to the nucleus had been largely completed before the seed plants evolved. However, a few relatively recent gene transfer events involving plastid ribosomal protein genes have also been described, including the transfer of *rpl22* to the nuclear genome in legumes (Gantt et al., 1991) and the transfer of *rpl32* to the nuclear genome in poplar (Bock, 2017; Ueda et al., 2007). A somewhat different, and particularly curious, case of plastid gene loss concerns the *rpl23* gene in spinach that has been replaced by a eukaryotic‐type L23 protein version that is encoded in the nuclear genome and imported into chloroplasts (Bubunenko et al., 1994). Interestingly, remnants of the *rpl23* gene are still found in the plastid genome of spinach, suggesting pseudogenization as an intermediate evolutionary step on the way to the complete loss of the gene (Bubunenko et al., 1994). Remarkably, the functional Rpl23 protein of the spinach ribosome does not come from a nuclear copy originating from endosymbiotic gene transfer to the nucleus. Instead, a nuclear gene encoding the homologous protein of the eukaryotic (80S) ribosome has been repurposed to replace the prokaryotic (chloroplast) Rpl23 protein (Bubunenko et al., 1994).

Another exception from the generally well‐conserved set of chloroplast‐encoded ribosomal proteins is the S16 protein of the 30S ribosomal subunit. Rps16 is an essential component of the chloroplast ribosome, as demonstrated by reverse genetic analysis in tobacco (Fleischmann et al., 2011). The *rps16* gene has been lost from the plastid genome multiple times in evolution. For example, it is absent from the plastid genomes of at least some gymnosperms (Tsudzuki et al., 1992), legumes (Doyle et al., 1995; Kato et al., 2000), members of the Salicaceae, Euphorbiaceae and Passifloraceae families (Alqahtani & Jansen, 2021), as well as several lineages of parasitic plants (Delannoy et al., 2011). In at least some of these cases, the loss of the chloroplast gene seems to have been facilitated by the acquisition of dual targeting properties by the nuclear gene encoding the mitochondrial S16 protein (Ueda et al., 2008).

The status of the *rps16* gene in the plastid genome of the model plant *Arabidopsis thaliana* is unclear. While some studies assume it to be a functional gene (e.g., Ueda et al., 2008), other studies have suggested loss of function due to the presence of a functional nuclear gene for the S16 protein (Roy et al., 2010). The recent development of a robust chloroplast transformation technology for *Arabidopsis* (Ruf et al., 2019, 2021) has made it possible to address the status of the *rps16* gene in the plastid genome of *A. thaliana* by reverse genetics. Here we report the generation of stable transplastomic *Arabidopsis* plants, in which we excised the *rps16* gene from the chloroplast genome. Comprehensive molecular and physiological characterization of the *rps16* knock‐out plants revealed no evidence for a relevant function in chloroplast translation. Together with the essentiality of the nuclear gene for the chloroplast S16 protein and the absence of detectable intron splicing from the plastid *rps16* transcript, our data suggest that (i) the functional S16 protein for the chloroplast ribosome is exclusively supplied by the nucleus, and (ii) the *Arabidopsis* chloroplast *rps16* is a pseudogene.

## RESULTS

### Targeted removal of the *rps16* gene from the *Arabidopsis* plastid genome

**Figure 1:**
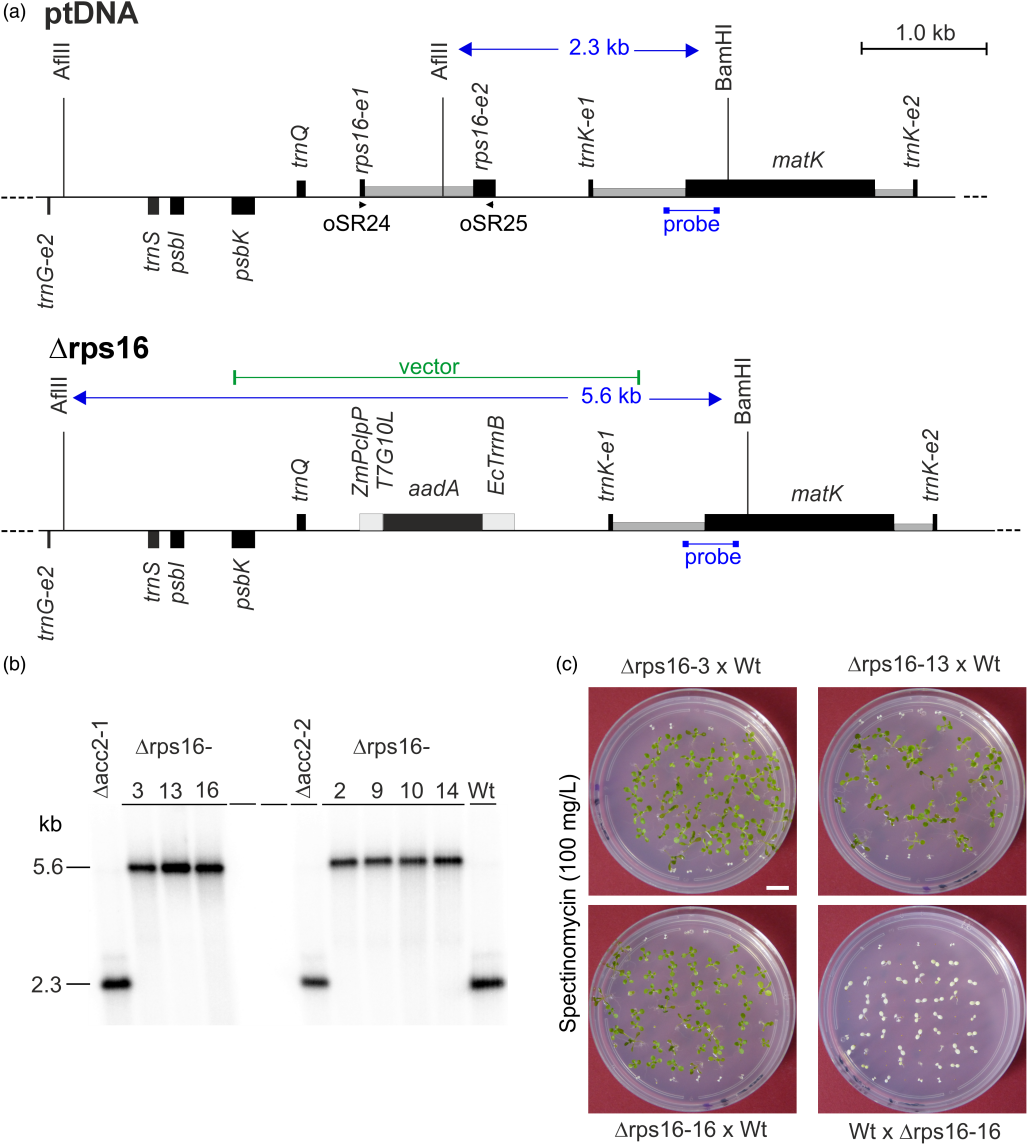
Targeted deletion of the *rps16* gene from the plastid genome of *Arabidopsis*. (a) Physical maps of the region in the *Arabidopsis* plastid genome (ptDNA; Sato et al., 1999) containing the *rps16* gene, and the transformed plastid genome in transplastomic Δrps16 knock‐out plants. Genes above the line are transcribed from the left to the right, and genes below the line are transcribed from the right to the left. The selectable marker gene *aadA* conferring spectinomycin resistance (Svab & Maliga, 1993) replaces the *rps16* coding region in the plastid genome of the Δrps16 plants. Restriction sites used for RFLP analysis are indicated. Expected sizes of restriction fragments detected by hybridization are given in kb. The hybridization probe used (derived from the *trnK*/*matK* locus) is indicated by the bar below the gene. The expression elements driving the *aadA* marker are shown as light gray boxes, and the introns in *trnK* and *rps16* are shown as lower dark gray boxes (exons are indicate by “e”). Oligonucleotides oSR24 and oSR25 were used as primers for RT‐PCR. (b) RFLP analysis of plastid transformants by Southern blotting. Digestion of DNA samples with the restriction enzymes AflIII and BamHI generates a 2.3 kb fragment in the wild type (Wt) and the recipient lines for chloroplast transformation (Δacc2; Ruf et al., 2019), and a 5.6 kb fragment diagnostic of the transplastome in all Δrps16 knock‐out mutants (cf. panel a). Seven independently generated transplastomic lines (Δrps16‐) are denoted by Roman numerals. Two independently generated Δacc2 lines (Ruf et al., 2019) were used for the chloroplast transformation experiments. Note that all seven transplastomic lines are homoplasmic and lack the 2.3 kb wild‐type‐specific hybridization band. The weakly cross‐hybridizing band above the 2.3 kb major band is most likely explained by the presence of promiscuous chloroplast DNA in the nuclear genome (Bock & Timmis, 2008; Hager et al., 1999). (c) Inheritance assays demonstrating the maternal inheritance of the spectinomycin resistance gene in a Δrps16 line and the lack of segregation in the progeny (confirming homoplasmy). As an internal control, each Petri dish contains two rows (top and bottom) of seedlings of the Δacc2 recipient line. All 47 germinated seedlings from the cross Δrps16‐3 x Wt, all 35 germinated seedlings from the cross Δrps16‐13 x Wt, and all 47 germinated seedlings from the cross Δrps16‐16 x Wt were green on spectinomycin‐containing medium, whereas all 40 germinated seedlings from the reciprocal cross Wt x Δrps16‐16 were white (i.e., sensitive to spectinomycin), consistent with homoplasmy and maternal inheritance of the plastid genome. The plates were photographed 15 days after sowing. Scale bar: 1 cm.

To functionally analyze the *rps16* locus in the *Arabidopsis* plastid genome, we used a reverse genetic approach that takes advantage of the recent development of a chloroplast transformation technology for *A. thaliana* (Ruf et al., 2019). To construct a knock‐out line, we cloned the *rps16*‐containing region of the *Arabidopsis* plastid genome into a plasmid vector and precisely replaced the *rps16* gene with an *aadA* expression cassette (Figure 1a). The *aadA* is a spectinomycin resistance gene that, due to the sensitivity of chloroplast ribosomes to the aminoglycoside antibiotic spectinomycin (Svab & Maliga, 1991), can be used as a selectable marker for plastid transformation (Bock, 2015; Svab & Maliga, 1993).

The knock‐out allele was then introduced into the *Arabidopsis* plastid genome of the Δacc2 recipient line for chloroplast transformation (Ruf et al., 2019) by particle gun‐mediated (biolistic) transformation, to replace the *rps16* locus with the *aadA* cassette by homologous recombination. Selection on spectinomycin‐containing medium (Ruf et al., 2021) produced multiple independent antibiotic‐resistant lines, seven of which were characterized (Figure 1b). Successful transformation of the plastid genome was confirmed via restriction fragment length polymorphism (RFLP) analysis by Southern blotting. Hybridization to a specific probe detecting the neighboring *matK* gene (Figure 1a) revealed the expected size shift in the chloroplast restriction fragment (Figure 1b). Moreover, the RFLP assay revealed the complete absence of the signal diagnostic of the wild‐type chloroplast genome (Figure 1b), suggesting that homoplasmy for the transformed plastid genome had been achieved. Given the high copy number of the plastid genome per cell (Greiner et al., 2020), the complete elimination of residual wild‐type copies of the genome is essential to obtain genetically stable transplastomic lines (Bock, 2015; Maliga, 2004). Homoplasmy of the transplastomic *rps16* knock‐out plants was ultimately confirmed by inheritance assays, in which seeds obtained from transplastomic T1 plants were germinated on spectinomycin‐containing medium. Consistent with the maternal inheritance of the plastid genome (Azhagiri & Maliga, 2007; Ruf et al., 2007), the Δrps16 mutants produced a uniform progeny of antibiotic‐resistant seedlings (Figure 1c), strongly suggesting that the knock‐out lines were homoplasmic.

### Phenotype of *Arabidopsis* Δrps16 mutant plants

In tobacco, *rps16* is an essential chloroplast gene that cannot be deleted from the chloroplast genome. Knock‐out attempts resulted in heteroplasmic plants that, in the absence of spectinomycin selection, quickly lost the transformed plastid genome copies (Fleischmann et al., 2011). Attainment of the homoplasmic state in our generated Δrps16 *Arabidopsis* plants (Figure 1) strongly suggested that *rps16* is not essential in *Arabidopsis*.

**Figure 2:**
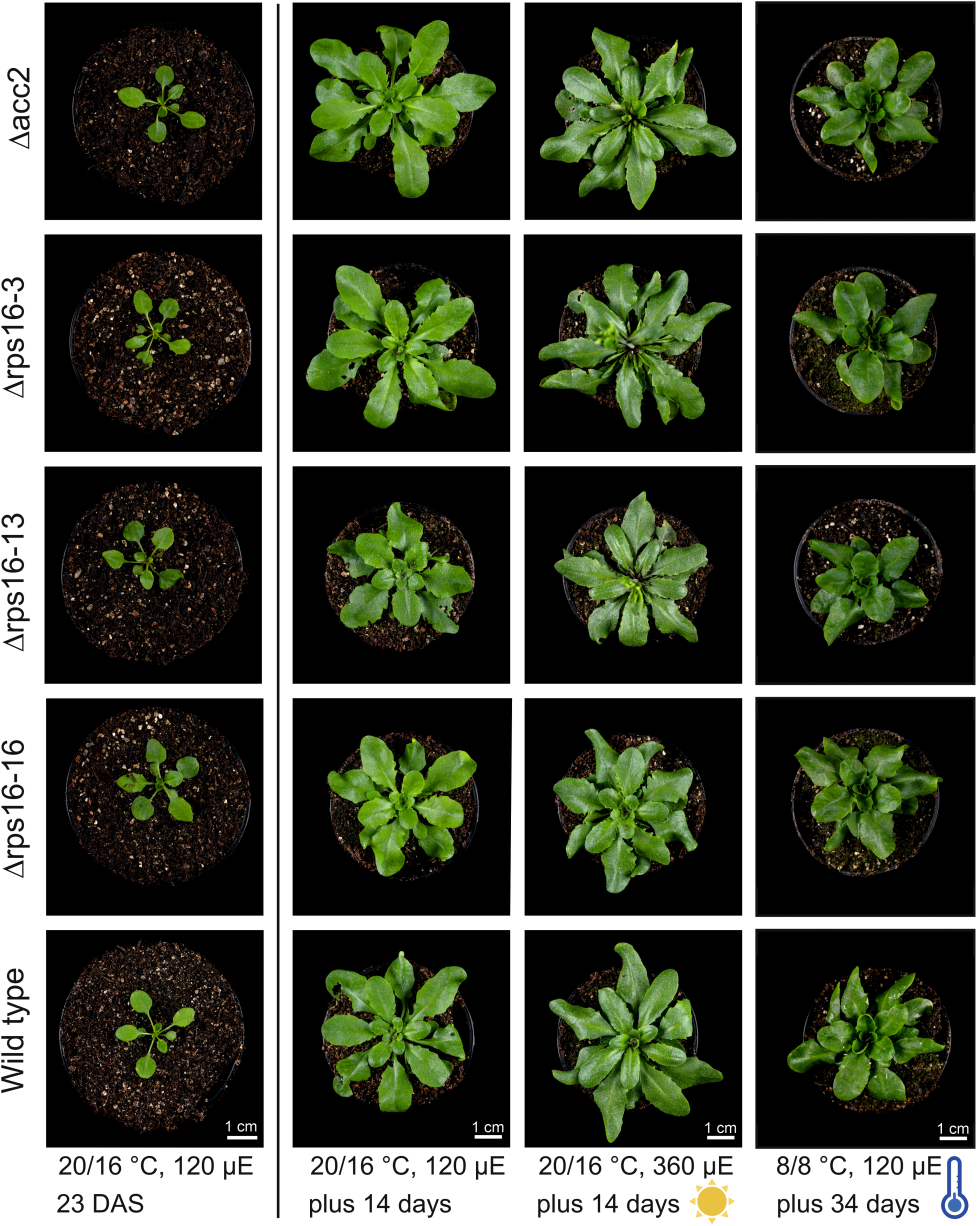
Phenotype of transplastomic Δrps16 mutants under different growth conditions. Three independently generated transplastomic lines (Δrps16‐3, Δrps16‐13, and Δrps16‐16) are compared with the wild type and the recipient line for *Arabidopsis* chloroplast transformation (Δacc2; Ruf et al., 2019). The growth experiments shown here involve the raising of plants from seeds under standard conditions (day temperature: 20°C, night temperature: 16°C; light intensity during the day: 120 μmol photons m^−2^ sec^−1^, here abbreviated as μE) for 23 days (left set of plants; DAS, days after sowing), followed by a growth period under different environmental conditions. Shown here are: continued growth under the nursery conditions for 14 days (second column), growth under high light for 14 days (360 μmol photons m^−2^ sec^−1^; third column), and growth under cold stress (8°C) for 34 days (right column). The photos show representative images of >15 plants raised per genotype and growth condition.

The phenotypes of the knock‐out plants were investigated in growth experiments with three independent transplastomic lines (Δrps16‐3, Δrps16‐13, and Δrps16‐16), the wild type, and the Δacc2 recipient line for *Arabidopsis* chloroplast transformation (Ruf et al., 2019). When grown under standard conditions (120 μmol photons m^−2^ sec^−1^, 20°C day temperature, 16°C night temperature, long‐day conditions), the knock‐out plants displayed no discernible phenotype (Figure 2). Since subtle disturbances of chloroplast gene expression can result in phenotypes that become apparent only under specific environmental conditions (e.g., Fleischmann et al., 2011; Petersen et al., 2011; Rogalski, Schöttler, et al., 2008), we also challenged the mutants with various stressful conditions, including exposure to high light (360 μmol photons m^−2^ sec^−1^, 20°C day temperature, 16°C night temperature) and cold stress (constant temperature of 8°C, light intensity during the day: 120 μmol photons m^−2^ sec^−1^). Previous work had shown that knock‐out mutants of some nonessential ribosomal proteins display phenotypes only under cold stress, but not under any other environmental condition tested (e.g., Fleischmann et al., 2011; Rogalski, Schöttler, et al., 2008), suggesting that low temperatures are particularly challenging when chloroplast ribosome function is compromised. However, none of the stressful conditions applied to the *Arabidopsis* Δrps16 mutants caused any phenotype that would be different from that of the control plants exposed to the same conditions (Figure 2). We, therefore, preliminarily concluded that the effect of *rps16* deletion from the plastid genome on chloroplast translational activity is minimal or non‐existent.

### Photosynthesis in Δrps16 mutant plants

**Figure 3:**
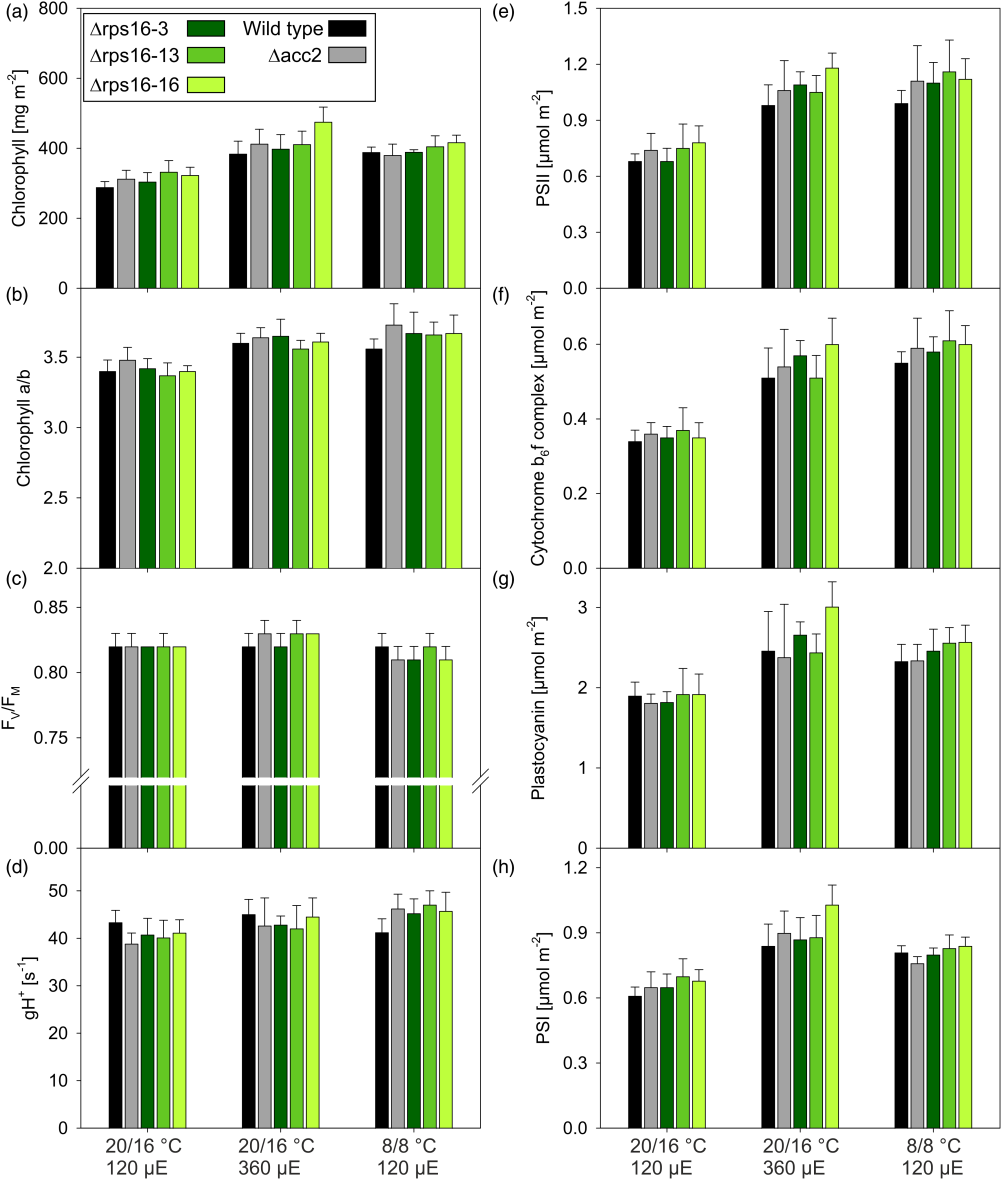
Analysis of chlorophyll contents and various photosynthetic parameters in *Arabidopsis* wild type (black bars), the Δacc2 recipient line (gray bars), and three independently generated Δrps16 mutants (three different shades of green). Plants were grown under standard conditions (20°C day temperature and 120 μmol photons m^−2^ sec^−1^ light intensity, left set), high‐light conditions (20°C day temperature and 360 μmol photons m^−2^ sec^−1^ light intensity, middle set), and chilling conditions (8°C day temperature and 120 μmol photons m^−2^ sec^−1^ light intensity, right set; see Materials and Methods section). μE, μmol photons m^−2^ sec^−1^. (a) Chlorophyll content per leaf area. (b) Chlorophyll‐*a*/*b* ratio. (c) Maximum quantum efficiency of PSII in the dark‐adapted state (F_V_/F_M_). (d) Thylakoid membrane conductivity of protons (gH^+^), a measure for the maximum activity of chloroplast ATP synthase. (e) PSII content per leaf area. (f) Content of the cytochrome *b*_6_ *f* complex per leaf area. (g) Plastocyanin content per leaf area. (h) PSI content per leaf area. Shown are average values with standard deviation. The number of independent biological replicates is *n* = 18 for panels a–d and *n* = 6 for panels e–h, except for the wild type under chilling conditions and the Δacc2 recipient line under high‐light conditions, for which the number of biological replicates is *n* = 21 (panels a–d) and *n* = 7 (panels e–h), respectively.

The efficiency of photosynthetic electron transport is a highly sensitive indicator of plastid translational capacity (Rogalski, Schöttler, et al., 2008). This is because most of the chloroplast genome‐encoded gene functions are directly or indirectly involved in photosynthesis (Bock, 2007). We, therefore, measured photosynthetic performance in wild‐type and transplastomic Δrps16 knock‐out plants. A number of photosynthesis‐related parameters were measured, including chlorophyll contents, the maximum quantum efficiency of photosystem II (F_V_/F_M_), the transthylakoid proton conductivity (gH^+^), and the contents of the major components of the photosynthetic electron transport chain in the thylakoid membrane (Figure 3). In addition, the light‐response curves of various chlorophyll‐*a* fluorescence parameters and functional parameters of photosystem I (PSI) were determined (Figures S1 and S2).

Photosynthetic parameters were first determined for plants grown under standard conditions (see Materials and Methods section for details). For none of the photosynthetic parameters measured, significant differences between the wild type, the recipient line Δacc2, and the three transplastomic knock‐out mutants were obtained. This was the case with the chlorophyll content per leaf area (Figure 3a), the chlorophyll‐*a*/*b* ratio, a proxy of the ratio of nucleus‐encoded antenna proteins (that bind both chlorophyll‐*a* and ‐*b*) to photosystems (that bind only chlorophyll‐*a*; Figure 3b), the maximum quantum efficiency of PSII in the dark‐adapted state (F_V_/F_M_; Figure 3c), and the activity of chloroplast ATP synthase (gH^+^; Figure 3d). The latter was determined from dark‐interval relaxation kinetics of the proton motive force across the thylakoid membrane. Also, the contents of PSII (Figure 3e), the cytochrome *b*_6_ *f* complex (Figure 3f), plastocyanin (Figure 3g), and PSI (Figure 3h) per leaf area did not reveal any consistent differences between the three mutant lines, the wild type, and the Δacc2 recipient line.

As no differences were seen under standard growth conditions, plants were next challenged with stressful conditions, including growth in the cold (8°C) and growth at increased light intensity (360 μmol photons m^−2^ sec^−1^; subsequently referred to as high light, HL). In the cold, metabolic consumption of ATP and NADPH is slowed down, while in HL, excitation pressure increases. Both treatments should result in an overreduction of the electron transport chain, especially the plastoquinone pool, and accordingly, in an increased rate of PSII photodamage. Thus, any impairment in chloroplast translation would be expected to result in increased PSII photoinhibition and, possibly, reduced accumulation of photosynthetic complexes, especially PSII, under these conditions.

However, similar to standard conditions, no consistent differences between the wild type, the Δacc2 recipient line, and the three Δrps16 mutant lines were detectable. As expected, under the stressful growth conditions, the photosynthetic apparatus showed pronounced acclimation responses in all plant lines. Both growth in the cold and in HL resulted in increases in the chlorophyll content per leaf area by 25–35% (Figure 3a). By contrast, changes in the chlorophyll‐*a*/*b* ratio were small, suggesting only minor reductions in the antenna size of PSII (Figure 3b). A reduced antenna size is a typical response of vascular plants to both high light and cold stress (reviewed, e.g., in Schöttler & Tóth, 2014). However, for *Arabidopsis*, it had previously been reported that, within a light intensity range from 100 to 400 μmol photons m^−2^ sec^−1^, only minor changes in the chlorophyll‐*a*/*b* ratio occur (Bailey et al., 2001). Also, F_V_/F_M_ was largely unaltered relative to the control conditions, suggesting that the plants did not suffer from photoinhibition (Figure 3c). ATP synthase activity (gH^+^) remained largely unaltered as well (Figure 3d); however, because this parameter is measured on a thylakoid basis, it increases per leaf area. This is because, both in the cold and in HL, the chlorophyll content and, therefore, the number of thylakoids per leaf area increased, so that proportional changes in total ATP synthase activity per leaf area occur. Among the photosynthetic complexes, PSII and the cytochrome *b*_6_ *f* complex were most strongly upregulated relative to control conditions, on average by 45–60%, respectively. Increases in plastocyanin and PSI were slightly less pronounced and in a similar range as those of the chlorophyll content per leaf area (Figure 3e–h).

Under control conditions, also the light‐response curves of chlorophyll‐*a* fluorescence parameters did not reveal any consistent significant differences between the wild type, the Δacc2 recipient line, and the three transplastomic mutants (Figure S1). Moreover, in line with the measured similar contents of the rate‐limiting cytochrome *b*_6_ *f* complex, no clear differences in light‐saturated electron transport capacity (ETRII) could be observed (Figure S1a). The light‐response curves of photoprotective non‐photochemical quenching (NPQ; Figure S1b) and the redox state of the PSII acceptor side (qL; Figure S1c) were also indistinguishable. Finally, Y(NO), a measure of non‐regulated dissipation of excitation energy in PSII (Kramer et al., 2004), did not differ between the wild type and the different mutants (Figure S1d). Similar to plants grown under control conditions, no clear differences could be observed for any of these parameters between the wild type, the recipient line Δacc2, and the three mutant lines under cold and HL conditions. However, in agreement with the increased cytochrome *b*_6_ *f* complex contents both in the cold and in HL, linear electron transport capacity was clearly higher under both conditions, relative to control conditions. In line with the higher electron transport capacity, the induction of NPQ was shifted to higher light intensities, and especially after growth in the cold, maximum NPQ capacity was increased. Similar to NPQ, the reduction of the PSII acceptor side (qL) was shifted to higher light intensities. Furthermore, due to the increased capacity for photoprotective NPQ, Y(NO) was reduced both after HL and cold acclimation in all plant lines. Likely, neither in the cold nor in HL did plants need to increase their PSII repair capacity because changes in electron transport capacity and in the capacity for NPQ were sufficient to compensate for the increased excitation pressure (in HL) and the slowed‐down metabolic activity (in the cold).

Finally, light‐response curves of the donor‐side (Y(ND)) and acceptor‐side limitation (Y(NA)) of PSI were measured (Schreiber & Klughammer, 2016; Figure S2a,b). Neither under standard conditions nor in the cold or in HL, significant differences between the wild type, the recipient line Δacc2, and the three mutant lines could be observed. In plants acclimated to the cold or to HL, the donor‐side limitation of PSI was shifted to higher light intensities, in line with the higher contents of both PSII and the cytochrome *b*_6_ *f* complex, and their higher electron transport capacity (Figure S1a). Also, the acceptor‐side limitation of PSI was not increased in either cold or HL, indicating that, in addition to the upregulation of electron transport capacity, the capacity of the downstream reactions of primary metabolism, and especially of the Calvin‐Benson cycle, was upregulated at least to a similar extent.

### Genome‐wide analysis of plastid transcript accumulation and translation in Δrps16 plants

**Figure 4:**
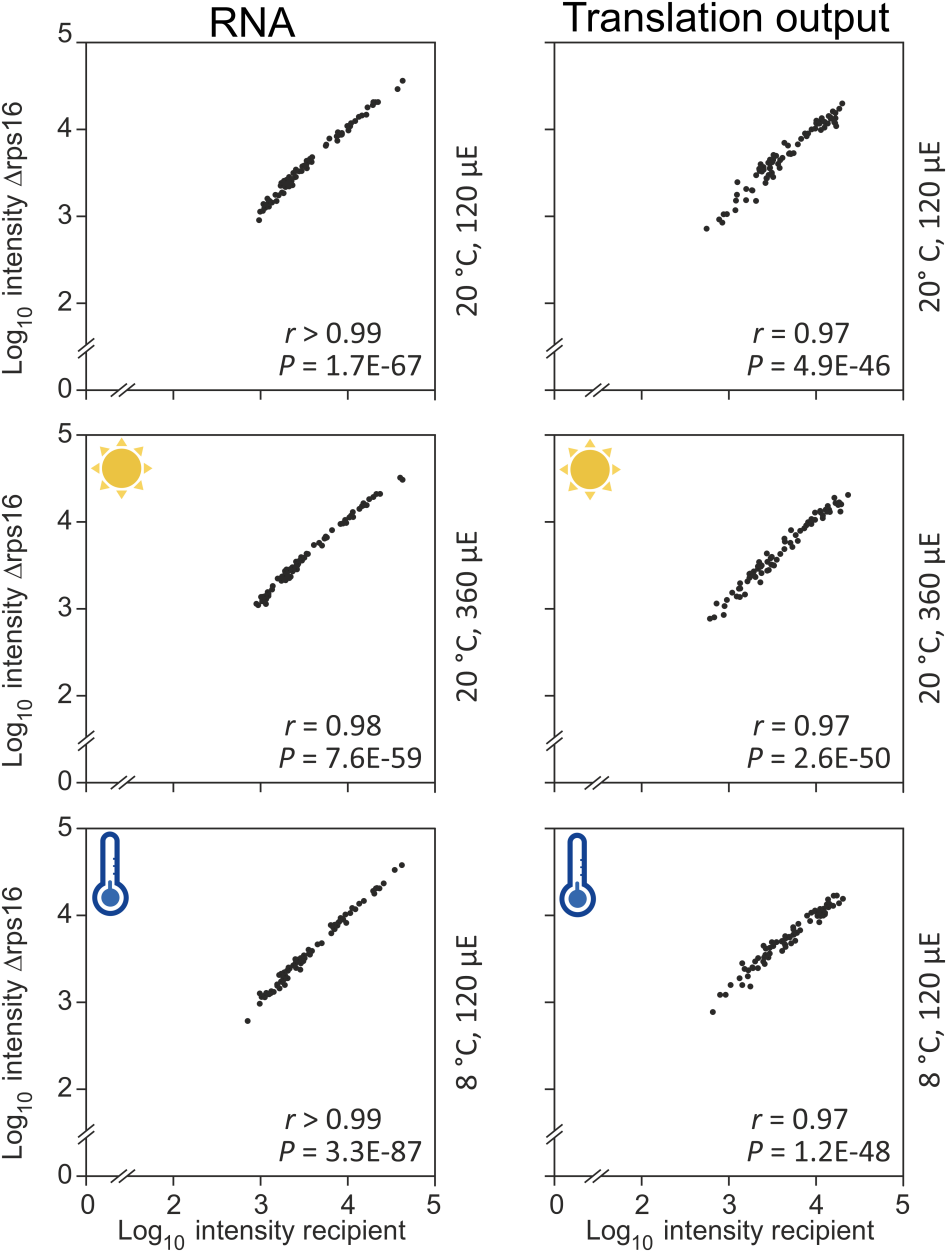
Genome‐wide analysis of plastid transcript accumulation (RNA) and translation output in *Arabidopsis* ∆rps16 mutants. Plants grown under standard conditions (upper row), and plants grown under high light (middle row) or cold stress (bottom row) were investigated (for growth conditions, see Figure 2). Relative plastid gene expression in ∆rps16 mutant plants was compared with that in the Δacc2 recipient line used for *Arabidopsis* chloroplast transformation (“recipient”; Ruf et al., 2019). For each plot, the mean of three biological replicates for the ∆rps16 mutant data were log_10_‐transformed and plotted against the respective data of the recipient line. For better visualization, axes are broken. Pearson's *r*‐values and ANOVA's *P*‐values are given within each plot (in nEm non‐superscript format) and demonstrate the very high similarity of plastid gene expression in the ∆rps16 mutants and the Δacc2 recipient line under different growth conditions. Note that none of the genes in the plastid genome show a more than two‐fold change in gene expression at either RNA accumulation or translation output level (Dataset S1). μE, μmol photons m^−2^ sec^−1^.

If the plastid *rps16* gene makes a relevant contribution to S16 synthesis, the Δrps16 knock‐out plants should show reduced levels of chloroplast translation. To directly investigate plastid translational activity in the knock‐out plants, comparative transcript and ribosome profiling experiments were conducted with recipient and mutant plants (Figure 4). In addition to growth under standard conditions, the two stress conditions HL and cold stress were investigated (cf. Figure 2). Consistent with the absence of a visual phenotype and any measurable difference in photosynthetic parameters, no significant differences in plastid transcript abundances were detected between the Δrps16 knock‐out plants and the control plants under any of the tested environmental conditions (Figure 4; left panels). Also, no significant differences were identified at the translational level, and the ribosome footprint numbers per reading frame were very similar for all plastid genes in the knock‐out mutants and the control plants (Figure 4; right panels; Dataset S1).

Taken together, our growth experiments under different environmental conditions, our measurements of photosynthetic parameters, and our analyses of mRNA abundances and translational output provided no evidence for any physiological defect or deficiency in plastid gene expression in the *rps16* knock‐out mutants.

### Lack of detectable intron splicing from *rps16* transcripts in *Arabidopsis*

The *rps16* gene contains a group II intron (Figure 1a), whose removal by splicing is likely required for the synthesis of a functional Rps16 protein product. Conflicting reports have been published on the efficiency of *rps16* intron splicing in *Arabidopsis*. Evidence for lariat formation has been obtained (Asakura & Barkan, 2006), and intron excision has been reported based on RT‐PCR results (Ueda et al., 2008). By contrast, another study, also employing RT‐PCR, reported a lack of splicing in *A. thaliana*, but correct intron excision in the closely related species *Arabidopsis arenosa* and *Arabidopsis lyrata* (Roy et al., 2010).

**Figure 5:**
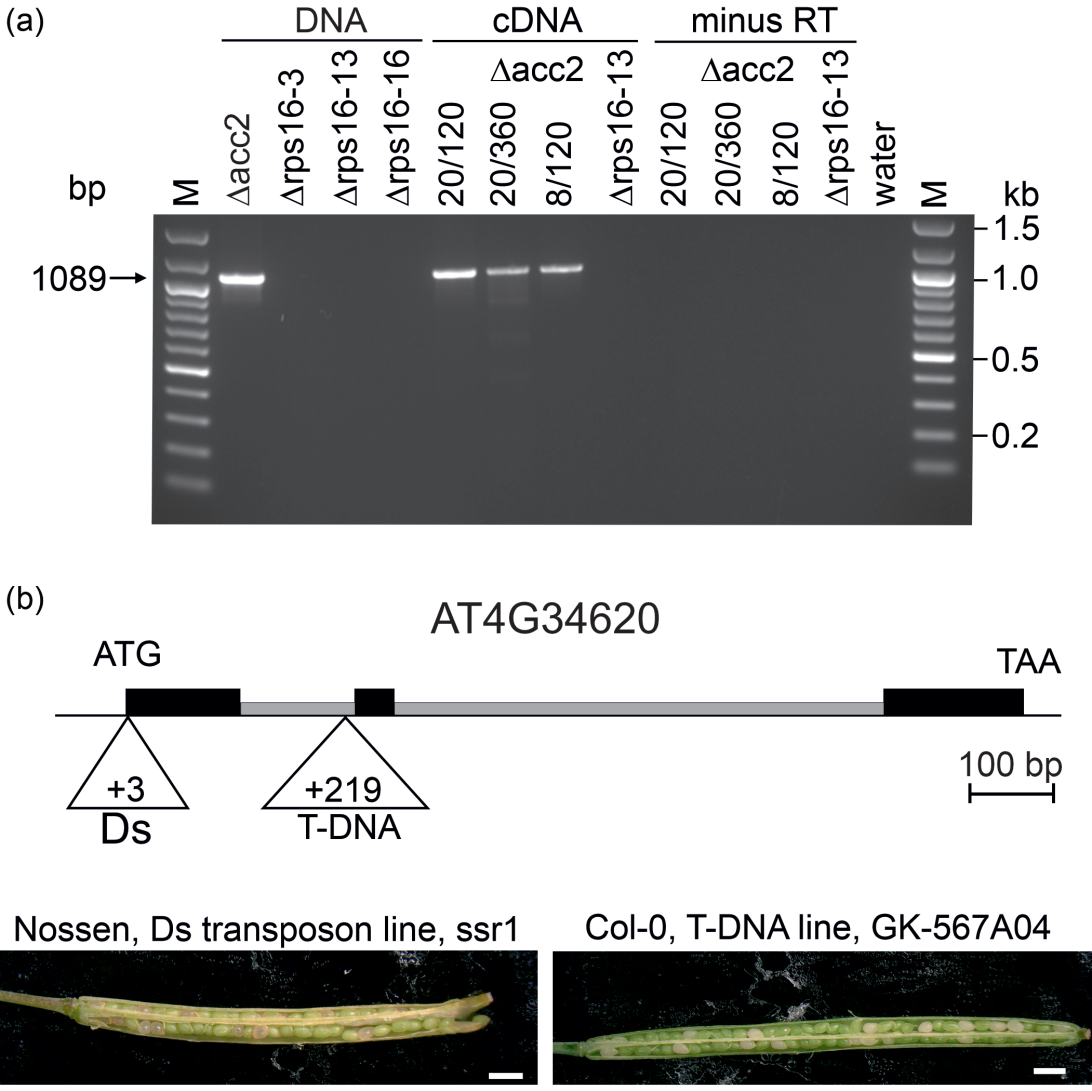
Lack of detectable *rps16* intron splicing and essentiality of the nuclear *RPS16‐1* locus in *Arabidopsis*. (a) Analysis of transcription and mRNA splicing of the *Arabidopsis rps16* gene. DNA, cDNA, and non‐reverse transcribed RNA (minus RT) from the Δacc2 recipient line for *Arabidopsis* chloroplast transformation (Ruf et al., 2019) and transplastomic Δrps16 lines were amplified with the primer pair oSR24/oSR25, combining a primer binding in exon 1 (*rps16‐e1*; cf. Figure 1a) with a primer binding in exon 2 of *rps16* (*rps16‐e2*; cf. Figure 1a; Table S1). The 1088 bp PCR product is obtained in both the DNA and the cDNA samples of the Δacc2 recipient line (but not in the transplastomic Δrps16 lines), suggesting that *rps16* is transcribed but not spliced. The PCR products were electrophoretically separated in a 1.3% ethidium bromide‐stained agarose gel. M: DNA size marker; water: buffer control. (b) Embryo lethality of homozygous insertion mutants for the nuclear *rps16‐1* gene (AT4G34620). A heterozygous Ds transposon insertion line in the ecotype Nossen and a heterozygous T‐DNA insertion line in the ecotype Col‐0 were investigated. The map shows the insertion sites of the Ds element and the T‐DNA, respectively. Exons are shown as black boxes, introns as gray boxes. Self‐pollinated heterozygous plants show segregation into viable (green in the immature silique) and inviable seeds (white) at a ratio of approximately 3:1, suggesting that the inactivation of *RPS16‐1* is embryo lethal. The Nossen allele was previously identified as embryo lethal (Tsugeki et al., 1996). Scale bars: 1 mm.

Given that our physiological and molecular analyses had provided no evidence for a function of the *rps16* gene in *A. thaliana*, we reinvestigated the splicing status of the *rps16* transcript. RT‐PCR assays with primers specific for the chloroplast *rps16* locus revealed clear signals for the unspliced transcripts that were completely absent from the Δrps16 knock‐out lines, as expected (Figure 5a). However, no signal for the spliced cDNA could be detected (Figure 5a). Given that the expected amplification product for the spliced cDNA is much smaller (224 bp) than that of the unspliced precursor (1089 bp), and smaller products have a strong amplification advantage in PCR, these data suggest that, if there is any intron excision from the *rps16* mRNA, its efficiency is very low.

### The nuclear *RPS16‐1* is an essential gene

It was previously suggested that the nuclear gene for the mitochondrial Rps16 protein has acquired dual targeting properties and supplies S16 protein also to the chloroplast (Ueda et al., 2008). A recent analysis of chloroplast and mitochondrial proteomes (van Wijk et al., 2024) has cast considerable doubt on this interpretation. Instead, the study identified two nuclear genes for organellar S16 proteins, referred to as *RPS16‐1* (described as encoded by locus AT4G34621, which is likely incorrect and, instead, should be AT4G34620) and *RPS16‐2* (AT5G56940). The proteomic data suggest that the *RPS16‐1* gene encodes the chloroplast S16 protein, whereas *RPS16‐2* encodes the mitochondrial S16 protein (van Wijk et al., 2024). These data raise the possibility that the previously suggested dual localization was an experimental artifact, possibly due to strong overexpression of the fluorescent reporter protein fused to the mitochondrial S16 version (encoded by *RPS16‐2*), which appears to be much lower expressed than the chloroplast‐targeted S16 protein (based on mass spectrometric peptide coverage; van Wijk et al., 2024).

The conflicting protein localization data notwithstanding, it seems clear that, in at least some plant species, a nuclear gene copy for the chloroplast S16 protein co‐exists with a functional *rps16* gene in the chloroplast (Ueda et al., 2008). If, in evolution, loss of the chloroplast gene function (by pseudogenization) would ensue, it would make the nuclear copy essential and the gene replacement essentially irreversible. Essentiality of nuclear genes is typically revealed by the embryo‐lethal phenotype of homozygous knock‐out lines (Bryant et al., 2011; Muralla et al., 2011). To test for essentiality of the nuclear *RPS16‐1* gene encoding the S16 protein that has been detected in chloroplast ribosomes of *Arabidopsis* (van Wijk et al., 2024), we investigated two mutants that carry insertions disrupting locus AT4G34620: a transposon insertion in the ecotype Nossen and a T‐DNA insertion line in the ecotype Columbia (Col‐0; Figure 5b). The insertion in the Nossen line was previously described as causing embryo lethality, but, based on the absence of a recognizable transit peptide sequence, the locus was proposed to encode a mitochondrial S16 protein (Tsugeki et al., 1996).

Following verification of the insertion sites in both insertion mutants by PCR assays, heterozygous mutant plants were allowed to self‐pollinate and the siliques were analyzed with respect to the presence of aborted seeds. Both lines segregated inviable seeds at the expected Mendelian ratio (Figure 5b), strongly suggesting that the nuclear *RPS16‐1* locus represents an essential gene. This finding also indicates that the *rps16* locus in the *Arabidopsis* plastid genome cannot functionally substitute for the *RPS16‐1* gene in the nucleus and is consistent with the strong evidence we have provided in this study for the plastid *rps16* being a pseudogene.

## DISCUSSION

The recent development of a robust chloroplast transformation protocol for the model plant *A. thaliana* (Ruf et al., 2019) has enabled the functional investigation of genes in the plastid genome by reverse genetics. In this study, we have applied the technology to conduct a knock‐out analysis of a chloroplast gene of unclear function. The S16 protein of the plastid ribosome of *Arabidopsis* and several other seed plant species had been reported to be potentially encoded by both a gene in the chloroplast genome and a nuclear gene. To clarify the functional status of the *rps16* gene in the *Arabidopsis* chloroplast genome, we generated *rps16* knock‐out plants by plastid transformation. Multiple lines of evidence support the conclusions that the nuclear *RPS16* locus is the only source of S16 protein in the plastid, and the plastid *rps16* gene is likely a transcribed pseudogene. First, homoplasmic *rps16* knock‐out plants can be readily isolated and show no visible phenotypes under a variety of growth conditions, including conditions that are particularly challenging to plants with mild plastid ribosome deficiencies (Figure 2). Second, the knock‐out mutants have no detectable physiological phenotypes (in their photosynthetic complex contents and functional parameters of light‐response curves of electron transport; Figure 3; Figures S1 and S2), and their plastid translational capacity is indistinguishable from that of wild‐type plants (Figures 3 and 4; Figures S1 and S2). Third, although the plastid *rps16* gene is expressed at the RNA level, we have been unable to detect spliced mRNA (Figure 5a). This finding contradicts previous studies that reported splicing of the group II intron residing within the *rps16* reading frame of *A. thaliana* (Asakura & Barkan, 2006; Ueda et al., 2008), but is in agreement with another study that reported lack of splicing in *A. thaliana*, possibly attributable to the mutated 5′ splice site of the group II intron (Roy et al., 2010). Given that the latter study, although detecting only unspliced mRNA in *A. thaliana*, found the intron to be spliced in the sister species *A. arenosa* and *A. lyrata* (Roy et al., 2010), it seems possible that there is natural variation in *rps16* splicing within the species *A. thaliana* in that some accessions still show some low‐level intron excision activity, whereas others have lost it completely. Finally, our analysis of the nuclear *RPS16‐1* locus confirmed that this gene is essential and its inactivation results in embryo lethality. It is important to note that the essentiality of the nuclear locus alone does not prove the non‐functionality of the plastid *rps16* locus, given that (i) the plastid and nuclear loci could be redundant, and (ii) the protein product of the nuclear locus has been suggested to be dually targeted and, therefore, its essentiality could be solely due to its function in mitochondria. However, recent proteomic data do not support the previously reported dual localization and, instead, suggest that one of the nuclear *Rps16* genes (*Rps16‐1*) serves the chloroplast, whereas the other (*Rps16‐2*) serves the mitochondrion (van Wijk et al., 2024). According to the recently proposed new nomenclature for ribosomal proteins, the gene product of *RPS16‐1* would be bS16c, and the gene product of *RPS16‐2* would be bS16m (Scarpin et al., 2023).

Interestingly, plastid targeting of a nuclear gene for the S16 protein (Ueda et al., 2008) was also reported for several other seed plants, including tomato (*Solanum lycopersicum*) and rice (*Oryza sativa*; Ueda et al., 2008), species that are believed to have a functional *rps16* gene in their plastid genomes. In light of our findings reported here, the functional status of *rps16* in other species may warrant reinvestigation. For example, in tomato, the *rps16* reading frame harbors a deletion of 10 nucleotides that causes a frameshift mutation (Kahlau et al., 2006). Since the site of the 10 bp deletion is close to the *rps16* stop codon, the resulting changes are restricted to the C‐terminus of the Rps16 protein (which is not well conserved among vascular plant species), and it was therefore assumed that the frameshift mutation could be functionally neutral (Kahlau et al., 2006). Whether or not this assumption is correct should be investigated by reverse genetics in tomato chloroplasts (Ruf et al., 2001) and, potentially, also in other species.

This work has exploited the recently developed chloroplast transformation technology for the model plant *A. thaliana* (Ruf et al., 2019) to analyze the function of the *rps16* gene in the chloroplast genome. As an alternative approach, the function of organellar genes can also be studied by expressing TALEN‐based genome editing reagents from the nuclear genome that are imported into the organellar compartment (Maliga, 2022; Tan et al., 2022). However, precise deletions are not possible to achieve with currently available genome editing tools for plastid and mitochondria (Forner et al., 2023; Kazama et al., 2019), and methods for site‐directed mutagenesis and base editing (Forner et al., 2022; Nakazato et al., 2022) suffer from substantial off‐target effects that occur in both the organellar and the nuclear genome (Lei et al., 2022). Thus, although genome editing offers a viable strategy for all systems in which organellar transformation is not yet possible, the unparalleled precision provided by homologous recombination remains the method of choice for reverse genetics in plant organelles.

In summary, our work presented here (i) has identified the *rps16* gene in the plastid genome of *Arabidopsis* as a transcribed pseudogene, (ii) suggests that gene transfer to the nucleus and evolution of the *RPS16‐1* locus have facilitated the functional deterioration of the plastid *rps16* locus, and (iii) indicates that the remaining *rps16* pseudogene represents an evolutionary intermediate on the way to the loss of the gene from the plastid genome of Brassicaceae.

## MATERIALS AND METHODS

### Plant material, growth conditions, and phenotypical assays

*Arabidopsis thaliana* ecotype C24 was used for all experiments on the chloroplast *rps16* gene reported in this study. For plastid transformation, the Δacc2 recipient line (Ruf et al., 2019) was employed and included in all subsequent experiments as an additional control. Plant material for chloroplast transformation was generated as described previously (Ruf et al., 2021).

For seed production and initial analysis of plant phenotypes, transplastomic plants were grown in soil under standard greenhouse conditions. Transgene inheritance was analyzed by germination of surface‐sterilized seeds on spectinomycin‐containing medium supplemented with 100 mg L^−1^ spectinomycin, as described previously (Ruf et al., 2021).

Growth tests under different environmental conditions were performed by raising wild‐type, Δacc2 and Δrps16 plants in soil under standard long‐day conditions in a controlled environment chamber for 23 days (day temperature: 20°C, night temperature: 16°C; light intensity during the day: 120 μmol photons m^−2^ sec^−1^, day length: 16 h; see Figure 2), followed by transfer to the stressful conditions.

Insertion mutants for the nuclear *RPS16‐1* locus (AT4G34620) were obtained from NASC, Nottingham, UK (Col‐0, GK‐567A04), and ABRC, Ohio State, USA (Nossen, ssr16).

### Construction of a plastid transformation vector for inactivation of *rps16*

A vector for the targeted knock‐out of *rps16* was constructed by replacing the *rps16* coding region with an *aadA* cassette driven by the *clpP* promoter from maize (Zhang et al., 2012), the *gene10* Shine‐Dalgarno sequence from coliphage T7, and the 3′ UTR from the *E. coli rrnB* gene (Ruf et al., 2019). To this end, the regions upstream and downstream of the *rps16* gene in the *Arabidopsis* plastid genome were amplified by PCR. The upstream homology region (nucleotide position 7188 to 6189; NC_000932.1) was amplified with primers oJF721 and oJF722, and the downstream homology region (nucleotide position 5083 to 4084) with primers oJF725 and oJF726 (Table S1). The chimeric *aadA* cassette was amplified with primer pair oJF723/oJF724 from plasmid pJF1153 (Ruf et al., 2019). The chimeric *aadA* is flanked by *loxP* sites. The three PCR products and the GreenGate entry vector pGGA000 (Lampropoulos et al., 2013) were digested with BsaI (a type IIS restriction endonuclease). Ligation of these four DNA fragments with T4 DNA ligase generated the final plasmid vector pJF1222 for chloroplast transformation.

### Plastid transformation and selection of transplastomic *Arabidopsis* lines

Plastid transformation experiments in *A. thaliana* ecotype C24 were conducted as described previously using the Δacc2 recipient line (Ruf et al., 2019, 2021). Briefly, microcalli induced from aseptically cultured *Arabidopsis* roots were bombarded with plasmid‐coated 0.6 μm gold particles using a helium‐driven biolistic gun (PDS1000He; BioRad). Spectinomycin‐resistant lines were selected on medium containing 50 mg L^−1^ spectinomycin (Ruf et al., 2021). Several independent transplastomic lines were produced, regenerated into plantlets, and grown to maturity as described previously (Ruf et al., 2021).

### Isolation of nucleic acids and hybridization procedures

Total DNA was extracted from *Arabidopsis* leaf material by a cetyltrimethylammoniumbromide (CTAB)‐based method (Doyle & Doyle, 1990). For RFLP analysis, DNA samples were digested with restriction enzymes, electrophoretically separated in 0.8% agarose gels, and blotted onto Hybond N nylon membranes (GE Healthcare). For hybridization, [α^32^P]dCTP‐labeled probes were generated by random priming (Multiprime DNA labelling kit; GE Healthcare). A radiolabeled PCR product was used as a hybridization probe. The PCR fragment was produced by amplification with primers oJF1034 and oJF1035 (Table S1). Hybridizations were performed at 65°C using standard protocols. Total plant RNA was isolated with the NucleoSpin^®^ RNA Plant Kit (Macherey‐Nagel) or, alternatively, by a guanidine isothiocyanate/phenol‐based method (peqGOLD TriFast; peqlab).

### cDNA synthesis and RT‐PCR

First‐strand complementary DNA (cDNA) was synthesized with the SuperScript III First‐Strand Synthesis System (Invitrogen) using 500 ng of RNA and random primer according to the manufacturer's protocols. PCR amplification was performed with the DreamTaq DNA polymerase (Thermo Scientific). *rps16* intron splicing was assayed with primers derived from the two exons of the gene (Table S1; Figure 1a).

### Transcript profiling and ribosome footprint analysis

Genome‐wide RNA profiling and ribosome footprint profiling for chloroplasts were performed using rosette leaves of Δrps16 mutant and control plants grown under three different conditions (cf. Figure 2) for RNA extraction and ribosome footprint isolation. The analyses were performed as described previously (Schuster et al., 2020; Trösch et al., 2018).

### *In vivo* measurements of photosynthetic parameters

For better comparability of functional data of control and cold‐exposed plants, all *in vivo* measurements of photosynthetic parameters were performed at 20°C. Light‐response curves of chlorophyll‐*a* fluorescence parameters were determined with the fiberoptics version of the DUAL PAM‐100 (Walz, Effeltrich, Germany). Leaves were dark‐adapted for 30 min prior to the measurement. Then, under light‐limited conditions, the light intensity was increased in 150 sec intervals. Under light‐saturated conditions above 500 μmol photons m^−2^ sec^−1^, the light intensity was increased every 60 sec. PSI‐related measurements were performed with the plastocyanin‐P_700_ version of the Dual‐PAM instrument as described previously (Schöttler et al., 2007). Plants were pre‐illuminated at growth light intensity for 3 min prior to measurements, to partially activate the Calvin‐Benson (CB) cycle and, in this way, avoid an acceptor‐side limitation of PSI. After 10 sec in the dark, the maximum difference absorbance signals of redox‐active plastocyanin and PSI were quantified by far‐red illumination for 8 sec, followed by a short saturating light pulse. Subsequently, the light intensity was stepwise increased, as described above for the chlorophyll‐*a* fluorescence measurements. The fraction of PSI reaction centers limited at the donor side, Y(ND), was determined according to published procedures (Schreiber & Klughammer, 2016). After the light‐response curves had been obtained, the chlorophyll content of the measured leaf section was determined in 80% (v/v) acetone (Porra et al., 1989).

The maximum amplitude of the electrochromic shift signal (ECS) was used as a measure of the light‐induced pmf across the thylakoid membrane. Leaves were pre‐illuminated for 6 min with saturating light (1295 μmol photons m^−2^ sec^−1^) to ensure that photosynthesis was fully activated, and ATP synthase activity was not limited by ATP consumption by the CB cycle. The saturating illumination was interrupted by 15 sec intervals of darkness, and the rapid initial phase of the relaxation kinetic of the ECS during the first 250 msec of darkness was fitted with a single exponential decay function to determine the time constant of the ECS decay. The reciprocal value, the thylakoid conductivity for protons (gH^+^), was used as a measure of ATP synthase activity. Multiple difference absorbance signals were simultaneously measured between 505 and 570 nm wavelength using the KLAS‐100 spectrophotometer (Walz, Effeltrich, Germany) and deconvoluted as previously described (Rott et al., 2011).

### Quantification of photosynthetic complexes

PSII, cytochrome b_6_f complex, and PSI were quantified spectroscopically in thylakoids isolated as previously described (Schöttler et al., 2004). PSII and cytochrome b_6_f complex contents were determined from difference absorbance signals of cytochromes *b*_559_ (PSII), *f*, and *b*_6_ (cytochrome *b*_6_ *f* complex) measured with a V‐750 photometer equipped with a head‐on photomultiplier (Jasco GmbH, Pfungstadt, Germany). After complete oxidation of all cytochromes by the addition of 1 mM potassium ferricyanide (+III), cytochrome *f* and the high‐potential form of cytochrome *b*_559_ were reduced by the addition of 5 mM sodium ascorbate, followed by the addition of 10 mM sodium dithionite to reduce the low‐potential form of cytochrome *b*_559_ and cytochrome *b*_6_. Difference absorbance spectra were calculated, baseline‐corrected, and deconvolved according to published procedures (Kirchhoff et al., 2002). PSI content was determined from difference absorbance signals of P_700_ as described (Schöttler et al., 2007). Plastocyanin contents, relative to PSI, were determined in leaves by *in vivo* difference absorption spectroscopy in the far‐red range of the spectrum and then recalculated based on the absolute P_700_ quantification in isolated thylakoids (Schöttler et al., 2007). Finally, all complex contents were re‐normalized to a leaf area basis with the known chlorophyll content per leaf area.

For statistical data analysis, Anova analyses using an “All Pairwise Multiple Comparison Procedure (Holm‐Sidak method)” were performed using SigmaPlot 14.5 (Systat). Even though a low threshold of *P* < 0.05 was used for the detection of statistically significant differences, not a single photosynthetic parameter of the three Δrps16 lines showed a consistent significant difference from either the wild type or the Δacc2 recipient line.

## CONFLICT OF INTEREST

The authors have not declared a conflict of interest.

## Supporting information


**Figure S1.** Light‐response curves of chlorophyll‐*a* fluorescence parameters of *Arabidopsis* wild type, the Δacc2 recipient line, and three independent Δrps16 mutants.
**Figure S2.** Light‐response curves of functional parameters of PSI of *Arabidopsis* wild type, the Δacc2 recipient line, and three independent Δrps16 mutants.

**Dataset S1.** Data from array‐based ribosome profiling experiments.

## Data Availability

Data supporting the findings of this work are available within the manuscript and its associated Supplementary Information.
