# Supplementary material for: Reverse genetics in the Arabidopsis chloroplast genome identifies rps16 as a transcribed pseudogene
Source: Plant J. 2025 May 7;122(3):e70198. doi: 10.1111/tpj.70198 (PMC12058238; doi:10.1111/tpj.70198)
Supplement: Supplementary file 1 — Figure S1. Light‐response curves of chlorophyll‐a fluorescence parameters of Arabidopsis wild type, the Δacc2 recipient line, and three independent Δrps16 mutants. Figure S2. Light‐response curves of functional parameters of PSI of Arabidopsis wild type, the Δacc2 recipient line, and three independent Δrps16 mutants. [file TPJ-122-0-s001.pdf]

Supplementary Materials

Supplementary Table 1. List of oligonucleotides used in this work. Recognitions sites for the restriction enzyme BsaI that were introduced for cloning purposes are underlined.

**Primer name**	**Target**	**Sequence 5' → 3'**
oJF721	*psbK*	AACAGGTCTCAACCTCAGCGGCTTGCCAAACAAAGGC
oJF722	upstream of *rps16* start codon (5' UTR)	TGTTGGTCTCTTTATAACATTCCTCTAGTTTGTGTAATTGATTC
oJF723	ZmPclpP, chimeric *aadA* in pJF1153	AACAGGTCTCAATAACTTCGTATAGCATACATTATACGAAGTTATTCTATGTATTAATAGAATCTATAGTATTC
oJF724	EcTrrnB, chimeric *aadA* in pJF1153	TGTTGGTCTCTGACTATAACTTCGTATAATGTATGCTATACGAAGTTATGTAGAAACGCAAAAAGGCCAT
oJF725	downstream of *rps16* stop codon (3' UTR)	AACAGGTCTCAAGTCTTACTAAAACGAAATGAAATTAATG
oJF726	*trnK* intron	TGTTGGTCTCTTGTTTATTTATTCACTTGACCCAAATTGG
oJF1034	*matK*	GGTTCACATCCTTAGTGGGA
oJF1035	*trnK* intron	AATCCGGTTAGCAGTCTACC
oSR24	*rps16-e1*	AAAACGATGTGGTAGAAAGC
oSR25	*rps16-e2*	TTCATTCCGTAAAAATCCCA

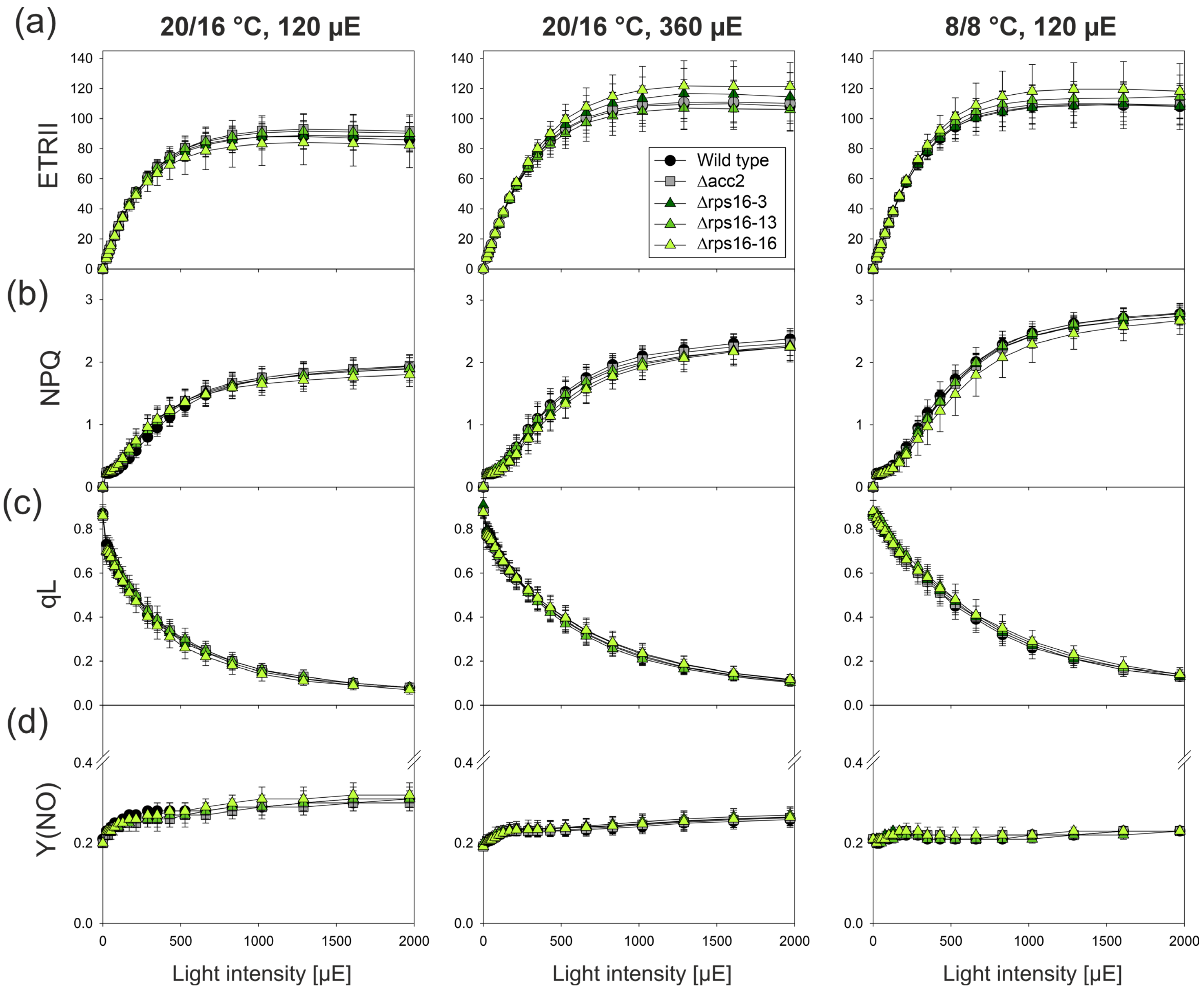

(a)
(b)
(c)
(d)
20/16 °C, 120 µE
20/16 °C, 360 µE
8/8 °C, 120 µE
ETRII
NPQ
qL
Y(NO)
Wild type
Δacc2
Δrps16-3
Δrps16-13
Δrps16-16
Light intensity [µE]
Light intensity [µE]
Light intensity [µE]

Supplementary Figure 1. Light response curves of chlorophyll-*a* fluorescence parameters of *Arabidopsis* wild type (black), the Δacc2 recipient line (grey) and three independent Δrps16 mutants (three different shades of green). Plants were grown under standard conditions (20°C day temperature and 120 µmol photons m^{-2} s^{-1} light intensity, left column), high-light conditions (20°C day temperature and 360 µmol photons m^{-2} s^{-1} light intensity, middle column) and chilling conditions (8°C day temperature and 120 µmol photons m^{-2} s^{-1} light intensity, right column; see Methods). µE: µmol photons m^{-2} s^{-1}. (**a**) Light response curves of linear electron transport (ETRII; in µmol electrons m^{-2} s^{-1}). (**b**) Light response curves of photoprotective non-photochemical quenching (NPQ), a measure for the regulated dissipation of excess excitation energy as heat. (**c**) Light response curves of qL, a measure for the redox state of the PSII acceptor side. (**d**) Light response curves of Y(NO), a measure for the non-regulated dissipation of excitation energy by PSII. Shown are average values with standard deviation. The number of independent biological replicates is n = 18, except for the wild type under chilling conditions and the Δacc2 recipient line under high-light conditions (n = 21).

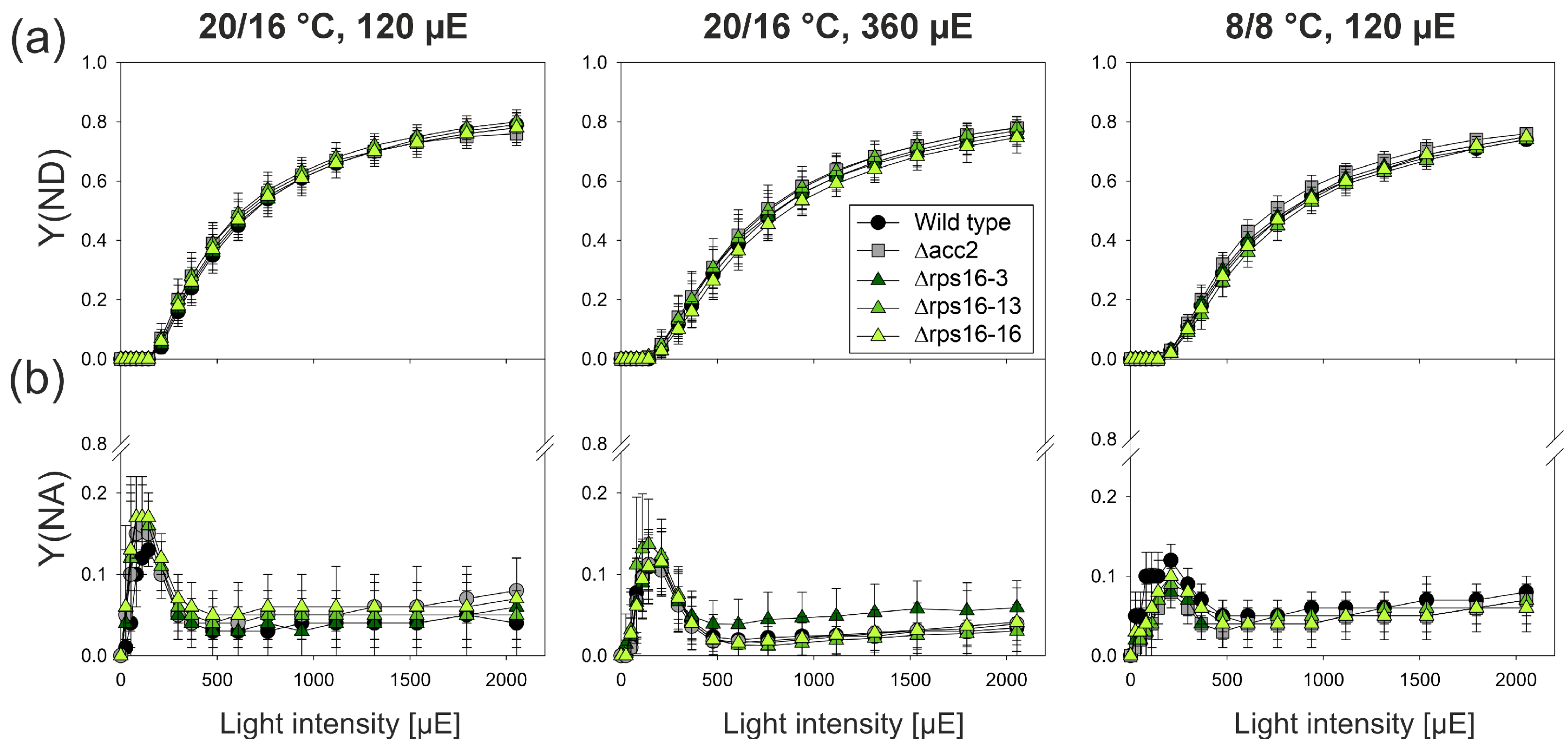

Supplementary Figure 2. Light response curves of functional parameters of PSI of *Arabidopsis* wild type (black), the Δacc2 recipient line (grey) and three independent Δrps16 mutants (three different shades of green). Plants were grown under standard conditions (20°C day temperature and 120 µmol photons m^{-2} s^{-1} light intensity, left column), high-light conditions (20°C day temperature and 360 µmol photons m^{-2} s^{-1} light intensity, middle column) and chilling conditions (8°C day temperature and 120 µmol photons m^{-2} s^{-1} light intensity, right column; see Methods). µE: µmol photons m^{-2} s^{-1}. (**a**) Light response curves of the donor-side limitation of PSI, Y(ND). (**b**) Light response curves of the acceptor-side limitation of

PSI, Y(NA). Shown are average values with standard deviation. The number of independent biological replicates is n = 18, except for the wild type under chilling conditions and the Δacc2 recipient line under high-light conditions (n = 21).

Supplementary Dataset 1. Data from array-based ribosome profiling experiments. Total RNA and ribosome footprints derived from Δacc2 recipient (rec) plants were labeled with Cy5, and the signals were detected at 635 nm. RNA and ribosome footprints derived from Δrps16 plants were labeled with Cy3, and the signals were detected at 532 nm. For details, see Methods section.
